# Large Doses of Calomel in the Treatment of Traumatic Tetanus

**Published:** 1867-08

**Authors:** J. J. Knott

**Affiliations:** Atlanta, Ga.


### ARTICLE IV.

*Large Doses of Calomel in the Treatment of Traumatic Tetanus.* By J. J. KNOTT, M. D., of Atlanta, Ga.

Private J. T. K——, Company C, 53d Georgia Regiment, Semmes' Brigade, McLaws' Division, A. N. Va., aged about thirty, dark hair, fair complexion, and by occupation, previous to inlistment, a farmer, received in the battle of Sharpsburg, Md., a gun-shot wound through the buttock—the ball in its passage, dividing the gluteal nerve. No untoward symptoms supervened, until the morning of the 27th of September, ten days after the reception of the wound, at which time the patient reported to me with the following symptoms: Frequent spasms of wounded limb, soreness, with contraction of muscles of nape of neck, difficult deglutition, and slight trismus, bowels constipated. Ordered ol. ricini f ℥ i., ol. terebinthæ f ʒ i., to be repeated in four hours, should the first dose not act freely upon the bowels.

Sept. 27th.—Saw the patient about 3 o'clock, P. M. Medicine has acted freely; symptoms of tetanus more prominent; spasms very severe and persistent; directed morphiæ sulphas gr. iv., (fiat chart No. 4) one every three hours; sinapisms from nape of neck to sacram.

10 o'clock, P. M.—Tetanic symptoms fully developed—Plurothotonus; the abdominal muscles of wounded side, conveying to the touch the impression of hard balls or lumps under the skin; continue the morphine increasing the doses half grain.

September 28th.—Spasm more frequent and severe; flow of saliva from mouth very profuse. Constant attention of the nurse necessary to keep the patient on his bed. Requested my friend, Dr. Morton, of Virginia, to see the case with me Owing to the almost hopeless condition of the patient, we decided on a heroic plan of treatment, viz: One drachm doses of calomel to be repeated every three hours.

10 o'clock P. M.—Has taken four doses. (In administering the calomel, the teeth were separated with a case knife, sufficient to deposit the calomel on the tongue). Symptoms about the same. No action on the bowels; decided to postpone further treatment until next morning.

29th.—Patient has been up frequently through the night, from the action of the medicine, the operations consisting of blood, mucous, and billious matter; decided improvement in all the symptoms.

12 o'clock M.—Medicine still acting severely; evacuations the same, though attended with more pain; more decided abatement of all the symptoms. Ordered sulph. magnesia ℥ ss.

5 o'clock P. M.—Since administering the salts, does not experience so much pain, when he goes to stool; slight ptyalism. Ordered chlorate potass ʒ ii., aqua fontana f ℥ x., tablespoonful every four hours.

Sept. 30th.—Patient badly ptyalised; tongue considerably swollen; mercurial fetor; ulceration of mouth, fauces, and tongue. Continue chlorate potassia, and the following wash to be used repeatedly through the day: Creasote f ʒ ss., aqua fontana f ℥ x., pulv. acaciae qs., fiat sol. From this date the patient had no further symptoms of tetanus, though at the time of his leaving the field infirmary, he was suffering severely from the effects of the mercury—so much so, indeed, that on his arrival at Fredrick City, Surgeon Morton, under whose care he was placed, found it necessary to leave him at this place in "*General Hospital*," where he remained under treatment for some three or four weeks before he was able to proceed to Richmond for exchange. He continued to suffer from the effects of the mercury for some twelve or eighteen months after this time, but finally regained his wonted health, under the use of the iodide of potassium. Having commenced a report of this case, with the view only, of laying the facts in connection thereof before your readers, I will not attempt to theorize as to the "*modus operandi*" of the remedy, but leave them to make their own deductions from the facts as they stand reported.

